# Two *Festuca* Species—*F. arundinacea* and *F. glaucescens*—Differ in the Molecular Response to Drought, While Their Physiological Response Is Similar

**DOI:** 10.3390/ijms21093174

**Published:** 2020-04-30

**Authors:** Katarzyna Lechowicz, Izabela Pawłowicz, Dawid Perlikowski, Magdalena Arasimowicz-Jelonek, Joanna Majka, Adam Augustyniak, Marcin Rapacz, Arkadiusz Kosmala

**Affiliations:** 1 Institute of Plant Genetics, Polish Academy of Sciences, Strzeszyńska 34, 60-479 Poznań, Poland; kmas@igr.poznan.pl (K.L.); dper@igr.poznan.pl (D.P.); jcho@igr.poznan.pl (J.M.); aaug@igr.poznan.pl (A.A.); akos@igr.poznan.pl (A.K.); 2 Department of Plant Ecophysiology, Institute of Experimental Biology, Faculty of Biology, Adam Mickiewicz University, 61-614 Poznań, Poland; arasim@amu.edu.pl; 3 Department of Plant Breeding, Physiology and Seed Science, University of Agriculture in Kraków, Podłużna 3, 30-239 Kraków, Poland; rrrapacz@cyf-kr.edu.pl

**Keywords:** antioxidant system, drought tolerance, *Festuca arundinacea*, *Festuca glaucescens*, forage grasses, photosynthesis

## Abstract

Impact of photosynthetic and antioxidant capacities on drought tolerance of two closely related forage grasses, *Festuca arundinacea* and *Festuca glaucescens*, was deciphered. Within each species, two genotypes distinct in drought tolerance were subjected to a short-term drought, followed by a subsequent re-watering. The studies were focused on: (*i*) analysis of plant physiological performance, including: water uptake, abscisic acid (ABA) content, membrane integrity, gas exchange, and relative water content in leaf tissue; (*ii*) analysis of plant photosynthetic capacity (chlorophyll fluorescence; gene expression, protein accumulation, and activity of selected enzymes of the Calvin cycle); and (*iii*) analysis of plant antioxidant capacity (reactive oxygen species (ROS) generation; gene expression, protein accumulation and activity of selected enzymes). Though, *F. arundinacea* and *F. glaucescens* revealed different strategies in water uptake, and partially also in ABA signaling, their physiological reactions to drought and further re-watering, were similar. On the other hand, performance of the Calvin cycle and antioxidant system differed between the analyzed species under drought and re-watering periods. A stable efficiency of the Calvin cycle in *F. arundinacea* was crucial to maintain a balanced network of ROS/redox signaling, and consequently drought tolerance. The antioxidant capacity influenced mostly tolerance to stress in *F. glaucescens*.

## 1. Introduction

Water deficit is one of the main environmental factors that affects plant growth and development, and significantly reduces the yield of many crop species. Considering rapid climate change, with global warming being the most noticeable, we can expect that periods of drought will occur more often than previously, even in temperate regions. Thus, recognition of cellular mechanisms conferring plant drought tolerance seems to be crucial, also, for yielding improvement in important crops [1].

Grasslands cover around 70% of the world’s agricultural areas and are mainly used for fodder purposes [2]. Forage grasses from *Lolium* and *Festuca* genera are among the most important species in temperate regions. Our previous studies revealed that *Festuca* species could be used as model plants to precisely dissect mechanisms of resistance to a wide range of abiotic stresses [3,4,5,6,7,8,9,10]. Allohexaploid *Festuca arundinacea* Schreb. (2*n* = 6x = 42) and tetraploid *F*. *arundinacea* var. *glaucescens* Boiss. (hereafter recognized as *F. glaucescens*) (2*n* = 4x = 28) are closely related forage grasses [11], with a relatively high potential to withstand periods of water shortage. In *F. arundinacea*, drought tolerance is mainly associated with drought avoidance, which relies on a development of deep root system, leaf rolling, and a rapid stomatal closure during the prolonged drought periods in the field conditions [10,12]. However, our earlier experiments revealed that *F. arundinacea* could also develop a metabolic adjustment strategy during short but severe drought periods [5,6]. On the other hand, *F. glaucescens* was described earlier as a species characterized mainly by a metabolism deceleration and a reduction of growth, associated with ‘a quiescence’ under drought conditions, followed by a further regrowth after stress cessation, which enables it to survive and to resume metabolism, following irrigation [13,14]. However, the knowledge about molecular basis of drought tolerance is, in fact, strongly limited for this species and requires detailed research.

Photosynthesis is among the primary processes affected by drought. Its efficiency can be restricted by both stomatal and non-stomatal mechanisms. Closing of stomata declines CO_2_ availability, while a reduction of the Calvin cycle efficiency was indicated to be crucial among the non-stomatal mechanisms of photosynthetic limitations [8,15,16,17]. Drought, and especially its initial phase, can also result in the oxidative burst, which is manifested by an overproduction of reactive oxygen species (ROS) [18,19], such as hydrogen peroxide (H_2_O_2_), hydroxyl radical (^•^OH), or superoxide anion radical (O_2_^•-^) being the most harmful for the cell environment [20]. In comfortable conditions, these molecules in lower amounts serve as important signaling compounds in numerous processes, associated with plant growth and development. Reactive oxygen species are also recognized as key factors involved in stress signaling [21,22]. However, a stress-induced overproduction of ROS can lead to damage of photosynthetic apparatus and disturbance of different metabolic pathways [22,23,24,25]. Furthermore, the enhanced production of ROS during drought may support lipid peroxidation, finally leading to damage of cellular membranes [26]. However, during evolution processes, plants developed ROS-scavenging enzymatic and non-enzymatic systems, which proved to be among the most crucial components of drought tolerance [18,27,28]. The enzymatic antioxidant system includes glutathione reductase (GR, EC 1.6.4.2), glutathione peroxidase (GPX, EC 1.11.1.9), L-ascorbate peroxidase (APX, EC 1.11.1.11), catalase (CAT, EC 1.11.1.6), and superoxide dismutases (SOD, EC 1.15.1.1). Superoxide dismutases are classified according to their metal cofactors, and among them, Fe-dependent superoxide dismutase (Fe-SOD), Cu/Zn superoxide dismutase (Cu/Zn-SOD), and manganese superoxide dismutase (Mn-SOD), are distinguished. Moreover, these enzymes contribute to the maintenance of cellular redox balance, especially during stress response [29,30,31]. Under water deficit, up-regulated expression of genes encoding the antioxidant enzymes both at transcriptional and post-transcriptional levels or/and an increase of enzyme activities, were observed [31,32,33].

In the field conditions, *F. arundinacea* and *F. glaucescens* revealed generally different survival strategies to cope with drought [13,14]. Herein, we hypothesize that under simulated soil water deficit conditions in pots, physiological response of *F. arundinacea* and *F. glaucescens* to drought will be similar, however, drought tolerance of each species will be developed by activating, at least partially, different molecular mechanisms, associated with photosynthetic performance and activity of cellular antioxidant system. Thus, the comprehensive research presented in this paper involved: (*i*) analysis of plant physiological performance, including water uptake (WU), abscisic acid (ABA) accumulation, electrolyte leakage (EL) and lipid peroxidation, gas exchange (stomatal conductance, transpiration and carbon dioxide assimilation), and relative water content (RWC) in leaf tissue; (*ii*) analysis of photosynthetic capacity (chlorophyll fluorescence; gene expression and protein accumulation of three Calvin cycle enzymes, plastid fructose-1,6-bisphosphate aldolase (pFBA, EC 4.1.2.13), crucial for the regeneration step of the cycle, and plastid phosphoglycerate kinase (pPGK, EC 2.7.2.3) and plastid glyceraldehyde-3-phosphate dehydrogenase (pGAPDH, EC 1.2.1.12), both crucial for the reduction step of the cycle; as well as the activity of protein marker of the Calvin cycle, pFBA); (*iii*) analysis of antioxidant capacity (ROS generation; gene expression, protein accumulation and activity of antioxidant enzymes, glutathione reductase (GR), glutathione peroxidase (GPX), L-ascorbate peroxidase (APX), catalase (CAT), and superoxide dismutases: Fe-SOD, Cu/Zn-SOD, and Mn-SOD).

## 2. Results

### 2.1. Genomic Discrimination and Species Identity of F. arundinacea and F. glaucescens

Karyotype analysis confirmed the identity of each analyzed here species and revealed that *F. arundinacea* and *F. glaucescens* differed in the number of chromosomes and distribution of rDNA loci. *Festuca arundinacea* genome comprised of 42 chromosomes and was allohexaploid, while tetraploid *F. glaucescens* genome comprised of 28 chromosomes. Fluorescent in situ hybridization (FISH) with 5S rDNA revealed six signals in chromosomes of the high drought tolerant (HDT) and the low drought tolerant (LDT) genotypes of *F*. *arundinacea* and four signals in both genotypes of *F*. *glaucescens* (Figure S1, red signals). In all the analyzed plants, 5S rDNA loci were located in interstitial parts of chromosomes. The results of FISH with 35S rDNA showed four signals in both genotypes of *F*. *arundinacea* and *F*. *glaucescens* (Figure S1, green signals). However, these signals were distributed in a different way in the two species. In *F*. *arundinacea* karyotypes, one pair of chromosomes with terminal positions of 35S rDNA and one pair with interstitial position of this sequence (Figure S1A,B), were observed. In *F*. *glaucescens* genotypes, only terminal distributions of 35S rDNA, were noticed (Figure S1C,D). Thus, each species presented distinct genomic structure with respect to a total number of chromosomes and numbers of particular rDNA loci, as described earlier by Thomas et al. [34].

### 2.2. Physiological Parameters

**Figure 1:**
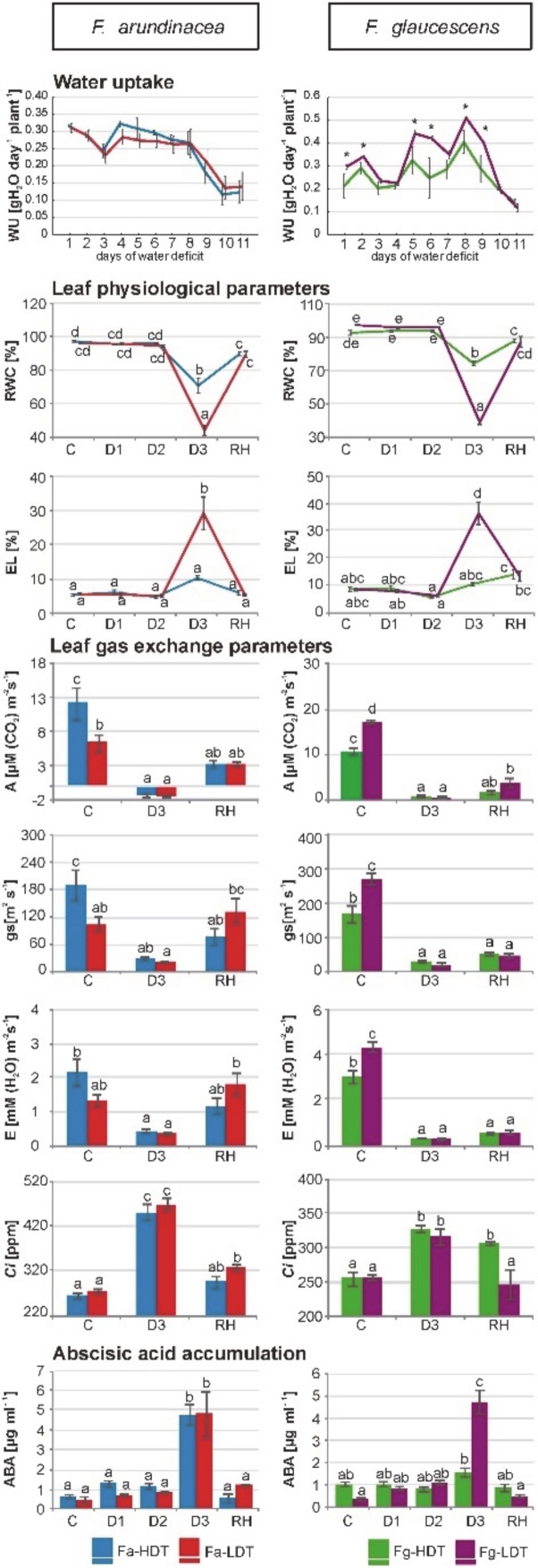
The water uptake (**WU**), relative water content (**RWC**), electrolyte leakage (**EL**), gas exchange parameters (CO_2_ assimilation (**A**), stomatal conductance (**g_s_**), transpiration (**E**), intracellular concentration of CO_2_ (***Ci***)) and accumulation of abscisic acid (**ABA**) in two genotypes of *F. arundinacea* (Fa-high drought tolerant (HDT), Fa-low drought tolerant (LDT)) and *F. glaucescens* (Fg-high drought tolerant (HDT), Fg-low drought tolerant (LDT)) during all water deficit or at different time-points: before stress treatment (C), on the 3rd (D1), 6th (D2), and 11th (D3) day of water deficit and 10 days after re-hydration initiation (RH). Error bars represent the standard errors (SE). Homogeneity groups are denoted by the same letters, according to Fischer LSD test (*p* = 0.01). Asterisks indicate differences between genotypes according to the student t-test (*p* = 0.05).

Under severe drought stage, a leaf rolling was noticeable in both genotypes of *F. arundinacea* and *F. glaucescens*. After rehydration, leaves returned to their normal shape in all the analyzed genotypes, nevertheless, numerous dried leaves could be also observed (Figure S2). In both genotypes of *F. arundinacea*, the level of WU was similar in the control and stress conditions. After a slight decrease in the initial phase of stress treatment, plants tried to maintain water absorption at the control level until more advanced time-points of drought period. However, the values of WU dropped significantly in both genotypes after eight days of water deficit and reached the lowest levels in the final steps of experiment (Figure 1). On the other hand, *F. glaucescens* revealed a different strategy of water uptake. Clear differences in WU were noticed between the *F. glaucescens* (Fg-HDT and Fg-LDT) genotypes in the control and more advanced stress conditions. Water uptake was higher in both genotypes after eight days of water deficit, compared with the control conditions, and then significantly dropped. During the experiment, at a majority of the analyzed time-points, WU was significantly higher in the LDT than in the HDT genotype of *F. glaucescens*. On the 11th day (D3) of drought, levels of WU in case of two *F. glaucescens* and two *F. arundinacea* genotypes, were similar (Figure 1). A significant decrease of RWC was observed on the 11th day (D3) of stress treatment, comparing to the control conditions (value 95–98%), in all the analyzed genotypes. However, in the LDT genotypes of both *Festuca* species (*F. arundinacea (*Fa)-LDT and Fg-LDT), RWC reduction was significantly larger (value around 40%) than in both HDT genotypes (Fa-HDT and Fg-HDT) (value around 70%). After further re-watering, RWC increased to the level observed in the control conditions in all the genotypes. Both, in the control conditions, initial phase of drought (D1, D2) and after stress cessation, there were no significant differences in RWC dynamics between the *F. arundinacea* and *F. glaucescens* genotypes (Figure 1). Values of EL did not change during the whole drought period in both HDT genotypes of two *Festuca* species but it significantly increased on the 11th day (D3) of water deficit in both LDT genotypes. Much higher EL increase was noted for the Fg-LDT genotype. However, after re-watering, EL values decreased to the control levels in two LDT genotypes (Figure 1).

Under severe drought (D3), both analyzed genotypes of *F. arundinacea* and *F. glaucescens* more or less reduced, to similar levels, their CO_2_ assimilation rate (A), stomatal conductance (g_s_), and transpiration (E) values; in case of the Fa-LDT genotype, a reduction of g_s_ and E was, however, not statistically significant. Under control conditions, higher A and g_s_ were reported for the Fa-HDT genotype and the Fg-LDT genotype. After re-watering, CO_2_ assimilation rate increased slightly but significantly only in the Fg-LDT genotype, while transpiration was higher, in comparison to the drought conditions, only in the Fa-LDT genotype (Figure 1). The intracellular concentration of CO_2_ (*Ci*) increased significantly in all the genotypes during the stress treatment. After further re-watering, a reduction of this parameter was observed in all the genotypes, except the Fg-HDT (Figure 1). Moreover, the values of some chlorophyll fluorescence parameters indicated significant differences between the HDT and LDT genotypes of both species, but only at D3 time-point of drought stress (Figure S3).

### 2.3. ABA Content

A content of ABA increased after 11 days of drought treatment (D3) in both *F. arundinacea* genotypes and in the LDT genotype of *F. glaucescens*. In the Fg-HDT genotype, there were no significant differences in ABA concentration during the whole period of stress treatment, as well as after recovery (Figure 1).

### 2.4. Expression of Genes Encoding the Calvin Cycle Enzymes at Transcript and Protein Levels

**Figure 2:**
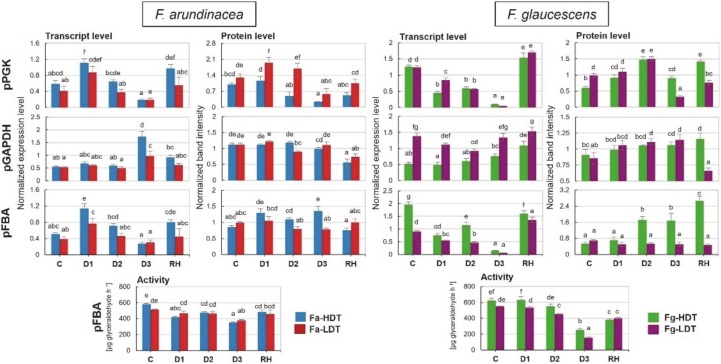
Transcript and protein accumulation level of plastid phosphoglycerate kinase (**pPGK**), plastid glyceraldehyde-3-phosphate dehydrogenase (**pGAPDH**), plastid fructose-1,6-bisphosphate aldolase (**pFBA**), and the activity of pFBA in two genotypes of *F. arundinacea* (Fa-HDT, Fa-LDT) and *F. glaucescens* (Fg-HDT, Fg-LDT) before stress treatment (C), on the 3rd (D1), 6th (D2), and 11th (D3) day of water deficit and 10 days after re-hydration initiation (RH). The transcript accumulation levels of actin and ubiquitin were used as references. Error bars represent the standard errors (SE) of three biological and two technical (transcript level and activity) or three biological (protein level) replicates. Homogeneity groups are denoted by the same letters, according to Fischer LSD test (*p* = 0.01).

Transcription patterns of pPGK and pFBA were similar in both *F. arundinacea* genotypes (Fa-HDT and Fa-LDT). After an initial increase of transcripts accumulation on the 3rd day of drought (D1), a subsequent decrease to the control levels, was observed. No changes in transcript accumulation, comparing to the control in the analyzed genotypes, were noticed after re-watering. However, clear differences between the genotypes (Fa-HDT and Fa-LDT) in pPGK and pFBA transcript levels were visible, after re-watering (RH) and on the 3rd day of drought (D1), respectively. The control level of pPGK transcript was equal in both *F. glaucescens* genotypes, whereas in case of pFBA, transcript level was about twofold higher in the Fg-HDT than in Fg-LDT genotype. Under water deficit, transcript level of both pPGK and pFBA significantly decreased in the Fg-HDT and Fg-LDT genotypes, achieving the lowest values on the 11th day of stress (D3). After re-hydration, pPGK accumulation level was significantly higher, compared to the control, in both genotypes, whereas pFBA level was higher only in the Fg-LDT genotype. Clear differences under stress conditions between *F. glaucescens* genotypes in pPGK and pFBA transcript abundance were observed at D1 and D2 time-point, respectively (Figure 2).

Transcript accumulation level of pGAPDH increased on the 11th day (D3) of water deficit, comparing to the control, in both *F. arundinacea* genotypes and it was significantly higher in the Fa-HDT genotype. After stress cessation, it returned to the initial level. In the HDT genotype of *F. glaucescens*, pGAPDH accumulation level remained relatively stable during the whole drought period, however, it increased about twofold after re-watering. In the Fg-LDT genotype, a slight drop of transcript level was observed at D2 time-point. During the whole experiment, level of pGAPDH transcript was higher in the Fg-LDT genotype, compared with the Fg-HDT genotype (Figure 2).

A significant increase of pPGK protein accumulation at the beginning of stress duration (between D1-D2) was observed in the LDT genotype of *F. arundinacea.* In the advanced drought (D3), it decreased to a lower level than that observed in the control conditions, while in the recovery phase, it returned again to the initial level. In the Fa-HDT genotype, pPGK protein level decreased in the late stage of stress (D2 and D3). An increase in accumulation of pPGK protein in both genotypes of *F. glaucescens* was observed during the first six days of drought duration. Then, its level decreased on the 11th day (D3) of drought and increased back after stress cessation in both genotypes. However, only in the Fg-HDT plant, the accumulation exceeded the control value (Figure 2).

No relevant changes in the pFBA accumulation patterns in the Fa-LDT genotype during the whole experiment, were observed. In the Fa-HDT genotype, a slight increase was noticed on the 3rd and 11th day of water deficit. However, that accumulation level dropped again after re-hydration to the values observed in the control conditions. The accumulation of pFBA protein in the Fg-HDT genotype increased from the initial time-points of drought duration (D1-D2) to the re-hydration time-point (RH). Interestingly, this level was constant during the whole experiment in the Fg-LDT genotype (Figure 2).

The amount of pGAPDH protein was more or less constant during drought treatment in two *F. arundinacea* genotypes, but it dropped after re-hydration. A similar tendency was observed for *F. glaucescens* genotypes, however, after re-watering, the amount of pGAPDH decreased only in the Fg-LDT genotype (Figure 2).

### 2.5. Activity of Chloroplast Aldolase

An activity of pFBA was more or less stable in *F. arundinacea* under control, drought, and recovery conditions. However, slight disturbances with respect to this parameter, were also observed during the experiment. The pFBA activity decreased slightly in both *F. arundinacea* genotypes in response to drought. In the Fa-HDT genotype, a slightly lowered activity was observed during the whole stress period, whereas in the Fa-LDT genotype only on the 11th (D3) day of drought. In contrary, in *F. glaucescens* genotypes, a significant deep decline of pFBA activity was observed between the 6th (D2) and 11th (D3) day of drought, compared to the control. After re-watering, an increase of pFBA activity, comparing to the advanced time-point of water deficit (D3), was revealed for both *F. glaucescens* genotypes, but the activity values were lower than the values observed in the control. The genotypes differed in pFBA activity during the whole stress period, and this activity was significantly higher in the Fg-HDT genotype (Figure 2).

### 2.6. Expression of Genes Encoding Antioxidant Enzymes at Transcript and Protein Levels

**Figure 3:**
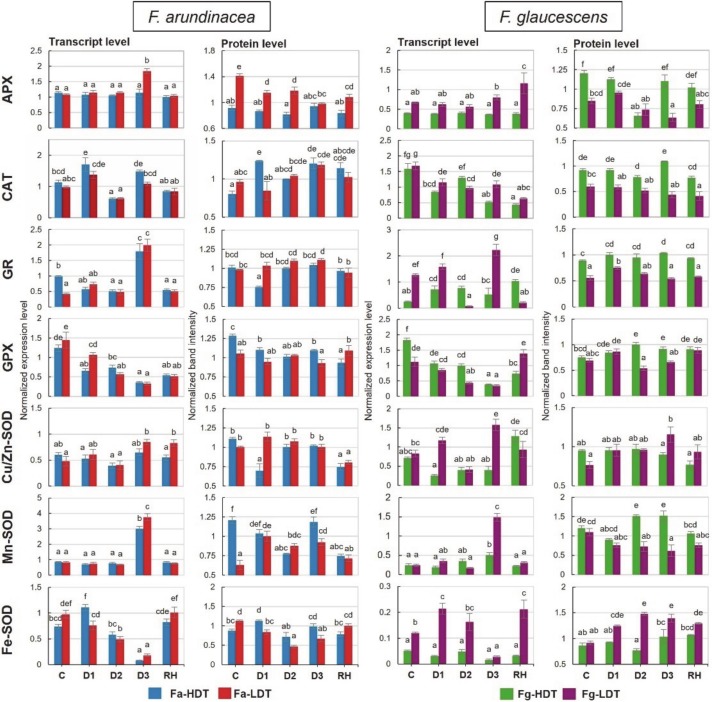
Transcript and protein accumulation level of L-ascorbate peroxidase (**APX**), catalase (**CAT**), glutathione reductase (**GR**), glutathione peroxidase (**GPX**), Cu/Zn superoxide dismutase (**Cu/Zn-SOD**), manganese superoxide dismutase (**Mn-SOD**), and Fe-dependent superoxide dismutase **(Fe-SOD**) in two genotypes of *F. arundinacea* (Fa-HDT, Fa-LDT) and *F. glaucescens* (Fg-HDT, Fg-LDT) before stress treatment (C), on the 3rd (D1), 6th (D2), and 11th (D3) day of water deficit and 10 days after re-hydration initiation (RH). The transcript accumulation levels of actin and ubiquitin were used as references. Error bars represent the standard errors (SE) of three biological and two technical (transcript level) or three biological (protein level) replicates. Homogeneity groups are denoted by the same letters, according to Fischer LSD test (*p* = 0.01).

A transcript accumulation of Cu/Zn-SOD did not change in the Fa-HDT genotype at all the analyzed time-points, while in the Fa-LDT genotype its level slightly increased at D3 and RH. In the Fg-LDT genotype, a reduction of Cu/Zn-SOD transcript accumulation was noticed at D2, while increased at D3 and dropped again, but only slightly, after re-watering. In the Fg-HDT genotype, changes in accumulation pattern occurred after re-hydration, and were associated with a significant increase of Cu/Zn-SOD transcript level (Figure 3). Protein accumulation of Cu/Zn-SOD was rather stable during the whole experiment in both *Festuca* species. Its slight reduction in the initial phase of stress duration (D1) and a higher accumulation, compared to control at D3, were observed in the Fa-HDT and Fg-LDT genotype, respectively. A lowering of its accumulation level was also noticeable in both *F. arundinacea* genotypes after re-hydration (Figure 3).

A higher transcript accumulation of Mn-SOD was observed on the 11th day of drought in both *F. arundinacea* genotypes and in the Fg-LDT genotype (Figure 3). In the control conditions, protein accumulation level of Mn-SOD was about twofold higher in the Fa-HDT genotype than in the Fa-LDT genotype. During the whole drought period, its level was elevated in the Fa-LDT, and then it dropped and returned to the control value after stress cessation. In the Fa-HDT genotype, a reduced protein accumulation of Mn-SOD at D2 and after re-watering, was noticed. A significant accumulation of Mn-SOD was observed between the 6th and 11th day (D2-D3) of stress duration in the Fg-HDT genotype. In the Fg-LDT genotype, a lowered protein level of Mn-SOD was remarked during the whole experiment in relation to the control (Figure 3).

On the 11th day of drought, a reduction of Fe-SOD transcript accumulation in both *F. arundinacea* genotypes as well as in the Fg-LDT genotype, compared with the control, was noted (Figure 3). The slight increase of Fe-SOD protein amount was observed only at the beginning of stress treatment (D1) in the Fa-HDT genotype. In the Fa-LDT genotype, a significant reduction in an amount of Fe-SOD protein during water deficit, was observed. After re-watering, it returned to the control level. In the Fg-LDT genotype, Fe-SOD protein was highly accumulated during the stress treatment as well as after regeneration (Figure 3).

An elevation of APX transcript accumulation was observed on the 11th day of stress treatment in the Fa-LDT genotype, as well as after re-watering, in the Fg-LDT genotype (Figure 3). Stress-induced changes in APX protein accumulation were noticed in the Fa-LDT genotype. Protein level was reduced, comparing to the control, during the whole drought period and after re-watering. A lowered APX accumulation was also observed at D2 in the Fg-HDT genotype and at D2 and D3 time-points in the Fg-LDT genotype (Figure 3).

Water deficit caused a reduction in accumulation of GPX transcript in both genotypes of *F. arundinacea* and *F. glaucescens*. A lower accumulation of GPX transcript was also maintained after re-hydration in two *F. arundinacea* genotypes, while in *F. glaucescens* genotypes its elevation was visible (Figure 3). At protein level, in the Fa-HDT genotype, a significant decrease of GPX was observed during drought treatment. In contrary, in the Fg-HDT genotype, accumulation of GPX was higher under stress treatment and after re-watering. In the Fg-LDT genotype, a decrease of its accumulation at more advanced time-points of drought (D2, D3), was observed (Figure 3).

An accumulation of GR transcript was higher on the 11th day of stress treatment in both *F. arundinacea* genotypes. On the other hand, an elevated level of GR transcript, compared to the control conditions, was observed in the Fg-HDT genotype at D1, D2 and RH time-points. A significant decrease of transcript accumulation was noticed in the Fg-LDT genotype at D2 and RH time-points (Figure 3). With respect to protein accumulation, on the 3rd day (D1) of water deficit a significant decrease of GR in the Fa-HDT genotype, was shown. This was the only time-point at which differences in GR amount between *F. arundinacea* genotypes, were revealed. On the other hand, a higher accumulation level of GR protein for the Fg-HDT, compared to the Fg-LDT genotype, was demonstrated during the whole experiment (Figure 3).

In the Fa-HDT genotype, a higher accumulation of CAT transcript was observed only in the initial stages of stress duration (D1). Both *F. arundinacea* genotypes reduced significantly transcript accumulation on the 6th day of drought but increased it again on the 11th day of drought. In *F. glaucescens*, a reduction in CAT transcript level was noticed during the drought period, as well as after re-watering in the Fg-LDT genotype and at D1, D3, and RH time-points in the Fg-HDT genotype (Figure 3). At protein level, no significant changes in CAT abundance during drought and after re-hydration was shown for the Fa-LDT genotype. In case of the Fa-HDT genotype, an amount of this protein was elevated at three time-points (D1, D3 and RH). Significant differences in accumulation of CAT between *F. glaucescens* genotypes were visible at all the experimental time-points, and this level was always higher in case of the Fg-HDT genotype (Figure 3).

### 2.7. Activities of Antioxidant Enzymes

**Figure 4:**
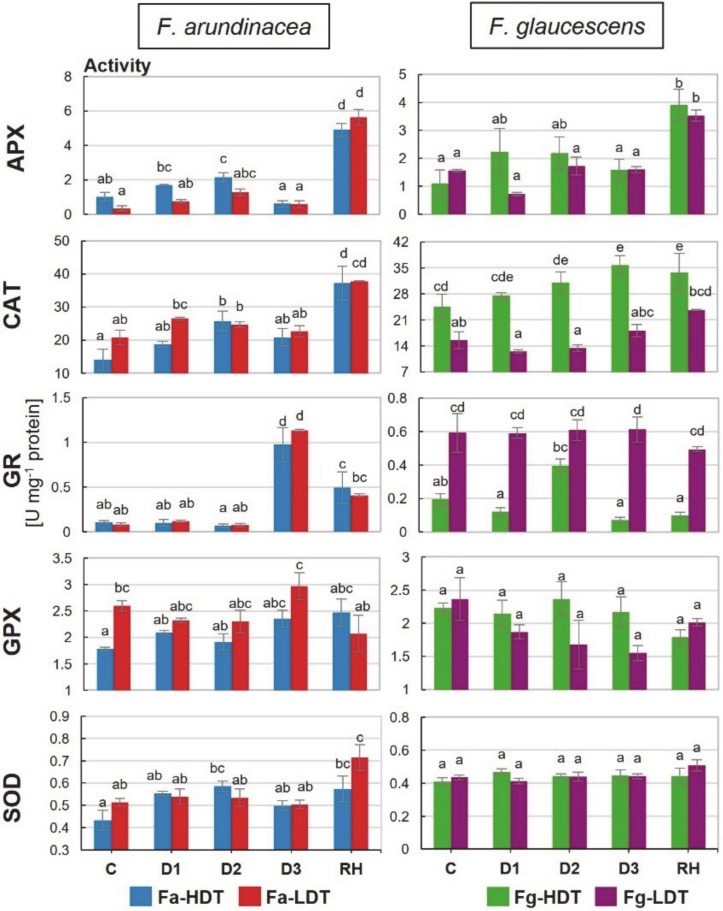
The activity of L-ascorbate peroxidase (**APX**), catalase (**CAT**), glutathione reductase (**GR**), glutathione peroxidase (**GPX**) and superoxide dismutase (**SOD**) in two genotypes of *F. arundinacea* (Fa-HDT, Fa-LDT) and *F. glaucescens* (Fg-HDT, Fg-LDT) before stress treatment (C), on the 3rd (D1), 6th (D2), and 11th (D3) day of water deficit and 10 days after re-hydration initiation (RH). Error bars represent the standard errors (SE) of three biological and two technical replicates. Homogeneity groups are denoted by the same letters, according to Fischer LSD test (*p* = 0.01).

No significant differences between *F. arundinacea* genotypes were observed in activities of all the analyzed here enzymes during drought and re-watering period. The only one difference was noticed in case of GPX at the control time-point, where its higher activity was revealed in the Fa-LDT genotype. In *F. glaucescens*, clear and significant differences in enzymatic activities were observed only for CAT, at all the time-points it was higher in the Fg-HDT, and for GR, at all the time-points, except D2, it was higher in the Fg-LDT genotype. An activity of GR increased in severe drought (D3) in both genotypes of *F. arundinacea* and at D2 time-point in the Fg-HDR genotype. In the Fg-LDT, no changes in GR activity during water deficit, was observed (Figure 4). A drought treatment and subsequent re-hydration did not lead to any significant changes in GPX activity in both *F. arundinacea* and *F. glaucescens* genotypes (Figure 4). A significantly higher APX activity was visible after re-watering in both LDT and HDT genotypes of two *Festuca* species. An increase in APX activity was also observed in the initial phase of water deficit (D2) in the Fa-HDT genotype (Figure 4). An activity of CAT was higher at D2 and RH time-points in the Fa-HDT genotype as well as at D3 and RH time-points in the Fg-HDT genotype, compared with the control conditions (Figure 4). A total SOD activity remained more or less constant in both genotypes of *F. arundinacea* and *F. glaucescens* in the control and drought conditions, but, compared to the control conditions, it increased significantly after re-hydration in both *F. arundinacea* genotypes (Figure 4).

### 2.8. Lipid Peroxidation

**Figure 5:**
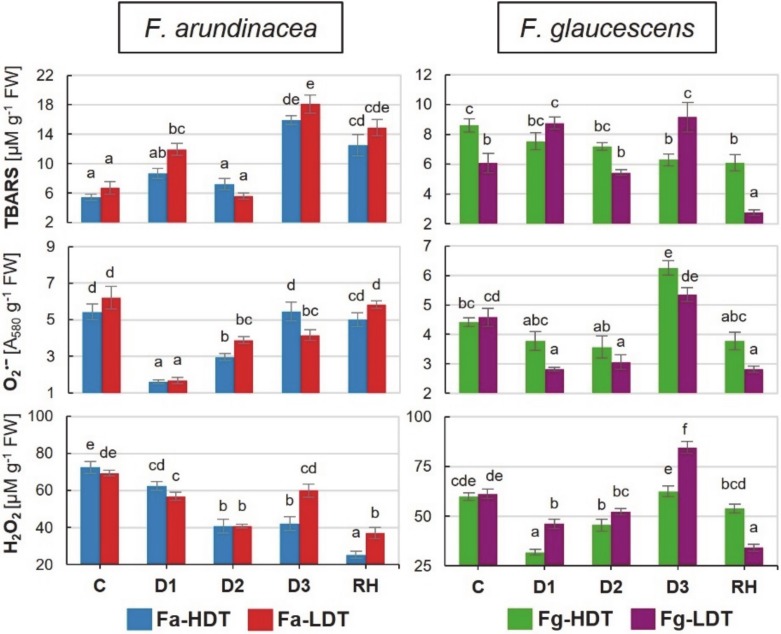
Thiobarbituric-reactive substances (**TBARS)**, superoxide anion radical (**O_2_^•−^**) and hydrogen peroxide content (**H_2_O_2_**) in two genotypes of *F. arundinacea* (Fa-HDT, Fa-LDT) and *F. glaucescens* (Fg-HDT, Fg-LDT) before stress treatment (C), on the 3rd (D1), 6th (D2), and 11th (D3) day of water deficit and 10 days after re-hydration initiation (RH). Error bars represent the standard errors (SE) of three biological and technical replicates. Homogeneity groups are denoted by the same letters, according to Fischer LSD test (*p* = 0.01).

Under water deficit, thiobarbituric-reactive substances (TBARS) accumulation increased significantly in both *F. arundinacea* genotypes. In case of Fa-HDT genotype, this phenomenon occurred on the 11th day of drought. In the Fa-LDT, an initial increase at D1 and a subsequent decrease of TBARS accumulation at D2 time-point, was noticed. Finally, the highest ca. threefold growth of TBARS level was observed on the 11th day of drought (D3) in both genotypes of *F. arundinacea*. Furthermore, this elevated level of TBARS was stable also after re-watering. In the Fg-HDT genotype, TBARS accumulation level showed a downward trend reaching the lowest value at D3 time-point. On the other hand, it increased significantly at D1 and D3 time-points in the Fg-LDT genotype, but dropped again, even below the level observed in the control conditions, after stress cessation (Figure 5).

### 2.9. Superoxide Anion Radical and Hydrogen Peroxide Contents

An amount of superoxide anion radical decreased drastically at the beginning of water deficit (D1), compared to the control, in both *F. arundinacea* genotypes. However, in the subsequent day of stress duration, its level started to rise. In the Fa-HDT genotype, it reached the value observed in the control on the 11th day of drought, whereas, in the Fa-LDT genotype, on the day 6th and 11th, it remained lower than in the control. A content of O_2_^•−^ did not change after re-watering in the Fa-HDT but increased more in the Fa-LDT. Contrary, in the *F. glaucescens* genotypes, a slight reduction of O_2_^•−^ amount at D1 and D2 time-points of water deficit was observed only for the Fg-LDT. In the advanced drought (D3), its content was significantly higher in both Fg-HDT and Fg-LDT genotypes, compared to the control conditions, however, without any significant differences between the genotypes. Both *F. glaucescens* genotypes revealed diminished accumulation level of this molecule after stress cessation (Figure 5).

A significant reduction of hydrogen peroxide accumulation between the control and the 6th day (D2) of drought was reported for both *F. arundinacea* genotypes. At the D3 time-point, this level was stable in the Fa-HDT genotype, whereas in the Fa-LDT, its value significantly increased to the control level. A decrease of hydrogen peroxide accumulation was observed for both *F. arundinacea* after stress cessation. A significantly higher accumulation of hydrogen peroxide was observed at D3 and RH time-points in the Fa-LDT, compared with the Fa-HDT genotype. The accumulation of H_2_O_2_ increased in the Fg-LDT genotype on the 11th day (D3) of drought. However, at the beginning of stress treatment (D1, D2) its level slightly decreased in both genotypes of *F. glaucescens*. Furthermore, a significant reduction in H_2_O_2_ generation was noticed also after stress treatment (RH) for these two genotypes but it was deeper in case of the Fg-LDT genotype (Figure 5).

## 3. Discussion

Plant tolerance to water deficiency involves morphological, physiological, and molecular adaptations. A majority of species are equipped with different survival strategies, which are activated depending on environmental conditions e.g., short periods of drought evoke different response than prolonged dehydration [15]. Our earlier research performed on *F. arundinacea* allowed us to recognize crucial components of chloroplast proteome [5], as well as roots architecture and metabolism [10], involved in drought tolerance of this species. In present research, other elements of leaf metabolic adaptations to drought conditions, associated with adjustments of carbon assimilation and enzymatic antioxidant system, were investigated in two closely related grass species, *F. arundinacea* and *F. glaucescens*. To the best of our knowledge, this is the first comprehensive research performed on the mechanisms of response to drought in *F. glaucescens*, at physiological and molecular levels. Furthermore, this is also the first detail comparison of stress response between *F. glaucescens* and *F. arundinacea*, concerning photosynthetic and antioxidant capacities of both species.

### 3.1. Physiological Response to Drought

A detail analysis of physiological parameters indicated that both *F. arundinacea* and *F. glaucescens* plants suffered from the applied conditions of water deficit. A higher decrease of leaf RWC during stress was observed in the LDT genotypes of both species, and it was accompanied by a relatively high increase of EL in these genotypes. However, these relations did not correspond with a dynamics of water uptake. Two *F. arundinacea* genotypes tried to maintain water absorption until the advanced time-point of drought period, while in the LDT genotype of *F. glaucescens* water uptake was more efficient in stress conditions than in the control. We can assume, first of all, that differences between *F. glaucescens* genotypes in the capacity to uptake water could be partially caused by differences in a mesophyll conductance [35] and also by a distinct root metabolism. Our recent study proved that in the case of *F. arundinacea*, a deep root system was crucial but not sufficient to avoid tissue dehydration under soil water deficit conditions. Furthermore, in the experiment performed in tubes, thus enabling undisturbed development of root system, not only architecture but also metabolic performance of roots were crucial to cope with negative effects of water shortage [10]. Second, this is also possible that the Fg-LDT genotype maintained a higher stomatal conductance in the early stages of drought (what was not measured in this experiment), and higher transpiration, which could trigger consequently the capacity of water uptake. Finally, we can also assume that higher disturbances in plant metabolism under drought conditions in the Fg-LDT genotype, could enhance water uptake in that plant at the early stage of stress duration. However, that mechanism was, in fact, insufficient for a longer time, and a drastic drop of water absorption, on the 11th day of drought period, occurred. This phenomenon was accompanied by a deep drop of RWC and a high increase of EL in the LDT genotype of *F. glaucescens*. Thus, although the dynamics of water uptake was different in two *Festuca* species, their physiological response to water deficit, including RWC and EL parameters, was in fact similar. A range of physiological differences observed between the LDT and HDT genotypes of both species, was also strikingly similar. Furthermore, after re-watering, the LDT genotypes of *F. arundinacea* and *F. glaucescens*, restored their leaf water relations and membrane integrity to the levels observed in the control conditions. This phenomenon indicates on the similar ability of both species to regenerate their leaf physiology, at least partially, after stress cessation. An increase of TBARS content was observed exclusively in *F. arundinacea* plants in the advanced drought period, and moreover, it remained at the elevated level, also after re-watering. However, in the Fa-HDT genotype, TBARS content did not correspond with a level of electrolyte leakage, which was significantly lower, compared with the LDT genotype of *F. arundinacea* on the 11th day of drought. A lack of correlation between membrane lipid peroxidation and membrane damage, expressed as a level of electrolyte leakage, was noted previously in *Portulaca oleracea* [36]. A considerable increase of lipid peroxidation caused by ROS accumulation under drought conditions was reported for many grass species, including *Glycine max* [37], *Triticum aestivum* [38], *Zea mays* [39], *F. arundinacea*, in the other research [40,41], and *Poa pratensis* [41].

Membrane disintegration and water relations were reversed after re-watering in both species, however, gas exchange parameters restitution seemed to be faster in *F. arundinacea*, as 10 days of irrigation was not sufficient to bring back the efficiency of stomatal conductance and transpiration in *F. glaucescens*. A high recovery capacity was reported in other grass species, like in a drought tolerant cultivar of *T. aestivum* [38], and inbred lines of *Z. mays* [42]. These observations demonstrated that a strategy of recovery after stress cessation could be also a dominant component of drought survival in pasture grasses, including *F. arundinacea*. Chlorophyll fluorescence parameters provide information about the efficiency of the flow of energy from an antenna to the electron transport chain components through the reaction center of photosystem II (PSII) [43]. Photochemical processes were more affected by drought in the Fa-LDT and Fg-LDT genotypes, compared to the HDT genotypes. However, values of all the denominated parameters of fluorescence returned to the control levels after stress removal in both LDR genotypes. This phenomenon confirmed again a high capacity to regenerate metabolism, after drought cessation, in *F. arundinacea* and *F. glaucescens*, and this regeneration ability was similar in both species, at least with respect to the applied environmental conditions (experiments in pots in the greenhouse).

A content of ABA increased in the advanced stage of stress in all the genotypes, except the HDT genotype of *F. glaucescens*, which revealed only slight, but statistically not significant change in the accumulation level of ABA during the whole period of drought. This could suggest that ABA-dependent drought signaling might be less important in this genotype or it was not initiated at the same time, as in *F. glaucescens* genotype of lower tolerance. On the other hand, the response to drought in the Fg-HDT was clearly visible in different physiological parameters, including RWC and gas exchange. This phenomenon was observed also in other crops, such as *Hordeum vulgare* [44]. Thus, despite similar physiological response to drought conditions and to further re-watering, we cannot exclude that *F. arundinacea* and *F. glaucescens* could, at least partially, activate different pathways of stress signaling. However, this aspect of our research requires further study to be fully explained.

### 3.2. ROS Production and Performance of Enzymatic Antioxidant System

An overproduction of ROS is a common phenomenon in plants exposed to different abiotic stresses, including drought. Maintaining a balance between their generation and scavenging plays a crucial role in a survival and growth restitution after cessation of adverse conditions. An increase of cellular antioxidant activity could protect plant cells against metabolism disturbances caused by abiotic stresses, including drought [45,46,47,48].

In *F. arundinacea* genotypes, superoxide anion radical and hydrogen peroxide were not accumulated to high levels under drought conditions and in the recovery phase. A decrease of H_2_O_2_ content in the Fa-HDT under stress conditions and after re-watering corresponded partially with the elevated CAT and APX activities. These results suggest that also other ROS particles could be involved in the peroxidation of membrane lipids, observed in *F. arundinacea* plants. A transient increase in GR activity noted in both *F. arundinacea* genotypes during water deficiency and a relatively high steady-state level of GR activity in the Fg-LDT genotype suggest that a pool of reduced glutathione (GSH) could play an essential role in the survival strategies of both *Festuca* species. An accumulation of non-enzymatic antioxidants, including GSH was crucial for ROS detoxification in drought tolerant genotype of *Amaranthus tricolor* [49]. Moreover, an improved GR activity that increased the supply of GSH for drought-induced ROS regulation was noted earlier in *Vigna radiata* [50]. An increased activity of GR in response to drought was also observed in other grass species, including *T. aestivum* [51], *T. durum* [52,53], and *Z. mays* [46]. After stress cessation, activities of APX, CAT, SOD, and GR in the Fa-LDT and Fa-HDT genotypes were elevated, compared to the control conditions, which is generally not a common phenomenon, since during re-watering, activities of antioxidant enzymes usually decrease [35]. One of the explanations for such the results could be a relatively short period of rehydration, applied in our study. One cannot exclude that a longer period of re-watering could result in a more effective turnover of enzymatic activities in this species. Moreover, different other studies underlined also the fact that a rate of recovery is the process strongly depended on severity of prior stress conditions [38]. Because *F. arundinacea* genotypes were not, in fact, exposed to the intense oxidative stress, their antioxidant response could be correlated with the overall strategy of drought recovery, clearly observed in this species. Furthermore, it was proved that a lipoxygenase-dependent lipid peroxidation can occur to form lipid hydroperoxides [54] used e.g., as substrates for drought-induced jasmonic acid synthesis. It should be noted that maintenance of low or moderate levels of ROS during stress conditions can facilitate their functions as the second messengers, mediating defense/tolerance reactions in plant cells, including stomatal closure or programmed cell death [55].

At the advanced time-points of drought, increased levels of superoxide anion radical in the HDT genotype, and hydrogen peroxide in the LDT genotypes of *F. glaucescens*, were observed. A content of H_2_O_2_ typically elevates gradually during prolonged drought, achieving higher levels in susceptible plants [35,38,42]. Such the situation was also observed in our experiment in the LDT genotype of *F. glaucescens*; hydrogen peroxide and TBARS levels increased at the advanced time-point of drought duration. Almost twofold higher activity of CAT in the control conditions in the Fg-HDT, compared with the Fg-LDT genotype, and its subsequent increase in the advanced drought, could be partially responsible for a lower level of hydrogen peroxide content and a lower level of membrane lipids peroxidation in the HDT genotype of *F. glaucescens*. This fact suggests that CAT plays a key role in H_2_O_2_ removal in *F. glaucescens*, similarly to other plant species [56]. This phenomenon also suggests that in *F. glaucescens*, drought induced oxidative stress was more intense than in *F. arundinacea*. Moreover, also protein accumulations of Mn-SOD, APX, GPX, GR, and CAT were significantly higher in the Fg-HDT genotype, than in the Fg-LDT genotype, both in the control and under stress conditions. These results indicate that the Fg-HDT genotype exhibited a higher antioxidant capacity, compared with the Fg-LDT genotype.

An accumulation of Fe-SOD in the Fg-LDT and Mn-SOD in the Fg-HDT genotype, might be associated with compensatory mechanisms to counteract enhanced superoxide anion radical production in response to drought stress in *F. glaucescens*. It was demonstrated earlier that SOD activity decreased in drought-susceptible cultivars of *T. aestivum*, exposed to stress conditions and, in contrary, maintained the control levels in drought-resistant cultivars of that species [52].

### 3.3. Efficiency of the Calvin Cycle

The Calvin cycle represents the important component of non-stomatal limitations of photosynthesis [8,17,57]. It is well-recognized that down regulation of photosystem II (PSII) activity results in an imbalance between the generation and utilization of electrons, apparently resulting in changes in quantum yield. These changes in photochemistry of chloroplasts in leaves of drought stressed plants result in a dissipation of excess light energy in the PSII core and antenna, thus cause a higher ROS generation, which is potentially dangerous under drought stress [58]. In the advanced stage of drought, gas exchange parameters were reduced in both *Festuca* species. An expression of the Calvin cycle enzymes was also affected by drought and significant differences between genotypes, were noticed. Three enzymes crucial for two different steps of the cycle were selected for the analysis—pPGK and pGAPDH, from the reduction step, and pFBA, from the regeneration step of the Calvin cycle. An up-regulation of transcript accumulation level of pGAPDH was revealed in *F. arundinacea* in the advanced drought, which was not visible in *F. glaucescens*. An expression of *TaGAPCp1* in leaves of *T. aestivum* seedling, exposed to drought (polyethylene glycol, PEG), was significantly up-regulated, peaking at 6 h of stress duration. Authors suggested that *TaGAPCp1* is a positive regulator of wheat tolerance to drought [59]. In contrary, PGK gene in *H. vulgare* exposed to 10 days of drought stress was significantly down-regulated [60]. However, generally, correlations between gene expression at transcript and protein levels, were shown to be relatively low for the enzymes analyzed here. This phenomenon indicates that in the regulation of the Calvin cycle performance, other components of cellular metabolism, including enzymatic activities *per se*, could be more important.

Plastid FBA was shown to be crucial for the performance of the Calvin cycle under drought conditions in our earlier experiment performed on *L. multiflorum/F. arundinacea* introgression forms. Mostly, its activity was proved to be a good indicator of the Calvin cycle efficiency [8,17]. Herein, we noticed species-specific differences with respect to this parameter, especially in its dynamics during the experiment. In case of *F. arundinacea*, pFBA activity was more stable during the control, drought and re-watering conditions, compared to *F. glaucescens*, though, a slight decrease of its activity was also noticed on the 11th day of drought. On the other hand, a higher accumulation of pFBA in the Fa-HDT and Fg-HDT genotypes in the advanced drought, and also a higher activity of pFBA on the 11th day of drought in the Fg-HDT genotype, were observed. Herein, we assume that a stable efficiency of the Calvin cycle during the stress conditions in *F. arundinacea*, manifested by a relative stable accumulation and activity of pFBA, could help to prevent a higher ROS (e.g., hydrogen peroxide) generation and accumulation. Furthermore, this stable efficiency could help also to maintain a balanced network of ROS/redox signaling, and consequently to develop drought tolerance of that species.

## 4. Conclusions

**Figure 6:**
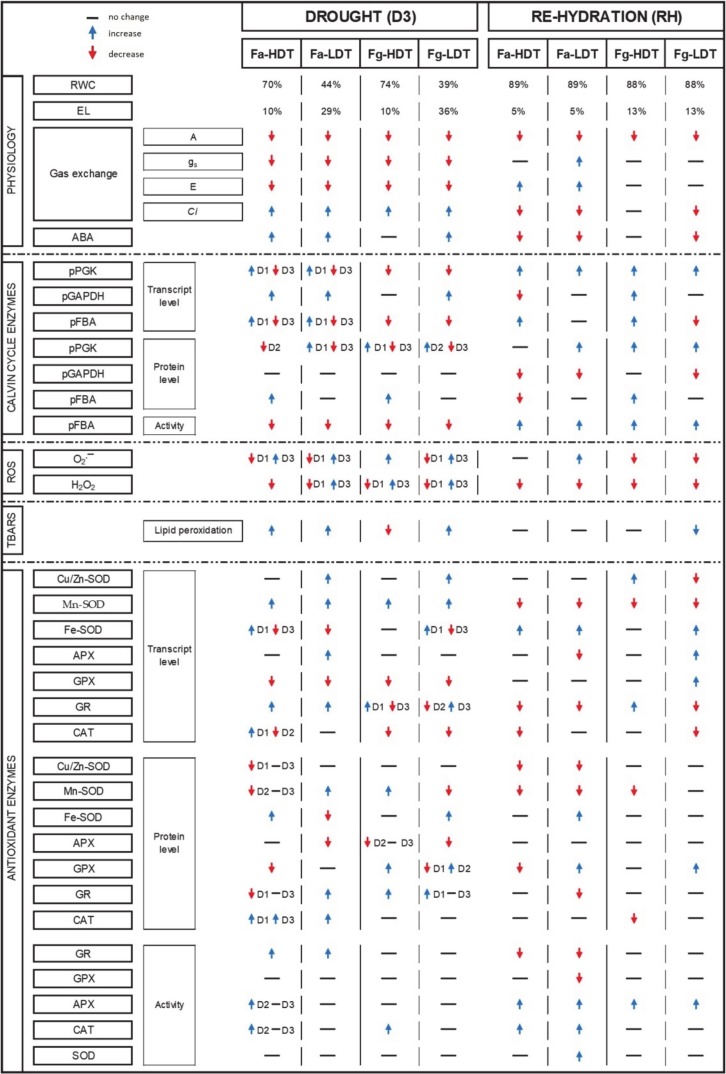
A comparison of physiological and molecular reactions in the HDT (Fa-HDT, Fg-HDT) and LDT (Fa-LDT, Fg-LDT) genotypes of *F. arundinacea* and *F. glaucescens* to drought stress on the 11th day of water deficit (D3), unless otherwise stated (D1—3rd and D2—6th day of drought) in relation to the control; and 10 days after re-hydration initiation (RH) in relation to D3.

Herein, we presented the research on two closely related grass species, *F. arundinacea* and *F. glaucescens*, under drought application in pots. Our analysis clearly showed that physiological reactions to water deficit, and subsequent re-watering, including alterations in a relative water content and membrane integrity, manifested by an electrolyte leakage, were similar in *F. arundinacea* and *F. glaucescens*. The HDT genotypes of both species were able to maintain water homeostasis and membrane stability under drought conditions, whereas the LDT genotypes revealed relatively high capacities to restore cellular homeostasis, after stress cessation. On the other hand, *F. arundinacea* and *F. glaucescens* revealed significant differences in the antioxidant and photosynthetic capacities under stress and re-watering conditions. We demonstrated that in *F. glaucescens*, drought induced oxidative stress was higher than in *F. arundinacea.* Consequently, an antioxidant capacity of *F. glaucescens* was proved to be a crucial component of drought tolerance in that species. This phenomenon was manifested mostly by higher accumulations of numerous antioxidant enzymes and also a higher CAT activity, observed in the genotype of higher drought tolerance. In contrary, we noticed that a stable efficiency of the Calvin cycle in *F. arundinacea* was crucial to maintain a balanced network of ROS/redox signaling, and consequently to develop drought tolerance of that species. Further research is required to compare the metabolism of both *Festuca* species in the field, or in the other conditions with enough soil space to develop roots, e.g., in tubes. All the performed analyses were presented in the scheme (Figure 6), which summarized the similarities and differences in crucial physiological and molecular components of drought response between the genotypes of *F. arundinacea* and *F. glaucescens*.

## 5. Methods

### 5.1. Plant Material, Growth Conditions, and Experimental Design

For the analysis, two genotypes within each species, *F. arundinacea* cv. Kord (2*n* = 6x = 42) and *F. glaucescens* (*F. arundinacea* Schreb. subsp. *Fenas* (Lag.) *Arcang*.) (2*n* = 4x = 28), significantly differing with their potential of drought tolerance: high drought tolerant (HDT) and low drought tolerant (LDT), were used (Table 1). Drought tolerance of selected genotypes was evaluated based on measurements of chlorophyll fluorescence (OJIP) during short-term drought treatment (11-day water deficit and further 10-day recovery) performed on pot-planted plants growing in the environmental chamber (hereafter termed pot-experiment) [5]. The genotypes of *F. arundinacea* derived from single seeds originated from the collection of Institute of Plant Genetics, Polish Academy of Sciences, created by Professor Zbigniew Zwierzykowski in 2010. Seeds of *F. glaucescens* (ABY-Bn 354-1980) derived from the collection of the Institute of Biological, Environmental and Rural Sciences (IBERS) (UK), originated from the Centre de Recherches de Lusignan, INRA (France) and donated in 1985 to IBERS. This collection at IBERS was held by the Genetic Resources Unit (Mr. Ian D. Thomas). The genotypes of *F. glaucescens* were obtained in 2014. The analysis of genomic structure was performed for both species, *F. arundinacea* and *F. glaucescens*, to precisely confirm their identity.

**Figure 7:**
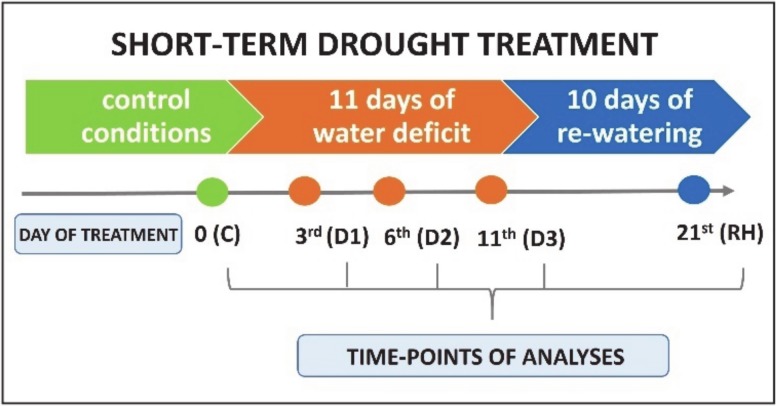
The scheme of short-term drought experiment performed with *F. arundinacea* and *F. glaucescens*.

Genotypes of both *Festuca* species were exposed for short-term drought in a pot-experiment as described previously by Kosmala et al. (2012) [5]. Each genotype was represented by 15 independent clones (each three growing in a separate pot with 4 dm^3^ of sand: peat (1:3) mixture). The conditions of experiment were as follows: temperature 22 °C, 16 h photoperiod, 400 μmol m^−2^s^−1^ photosynthetic photon flux density (PPFD), air humidity 50–60%. The plant material (leaf tissue) for the analysis was harvested before stress treatment when plants were fully hydrated (control, C), on the 3rd (D1), 6th (D2), and 11th (D3) day of watering cessation, and 10 days after subsequent re-watering (RH) (Figure 7). The experimental conditions and analyzed time-points, including controls, were the same as described in our earlier studies [5,8,17].

### 5.2. Fluorescent in Situ Hybridization (FISH)

To verify a genomic status of *F. arundinacea* and *F. glaucescens* plants, FISH experiment with two highly conserved rDNA sequences (5S and 35S rDNA) as probes was applied. Wheat clone pTa794 containing 5S rDNA was labeled by PCR with tetramethyl-rhodamine-5-dUTP (Roche, Mannheim, Germany). Whereas, 35S rDNA, generated from a 2.3 kb fragment of the 25S rDNA coding region of *Arabidopsis thaliana*, was labeled by nick-translation with digoxigenin-11-dUTP (Roche, Mannheim, Germany). Preparation of slides, labeling of probes and FISH experiments were performed according to protocols described by Majka et al. (2017) [61]. Briefly, in FISH protocol, a hybridization mixture consisted of 50% formamide, 2 × SSC, 10% dextran sulfate and 100 ng of rDNA probes. Hybridization mixture together with good quality of chromosome slides were denatured at 80 °C for 2 min and then incubated overnight at 37 °C. In the protocol, fluorescein isothiocyanate-conjugated (FITC) anti-digoxigenin antibody was applied to detect digoxigenin-labeled 35S rDNA probe. After counterstaining with 4, 6-diamidino-2-phenylindole (DAPI, Sigma, St. Louis, Missouri), slides were mounted in antifade Vectashield solution (Vector Laboratories, Burlingame, CA, USA). Slides were evaluated under an Olympus BX 61 automatic epifluorescence microscope equipped with an Olympus XM10 CCD camera. All the images were captured using Olympus Cell-F imaging software (ver. 3.1; Olympus Soft Imaging Solutions GmbH, Germany) and Micrographx Picture Publisher software (ver. 10; Corel Corporation, Canada).

### 5.3. Physiological Parameters

Water uptake, relative water content (RWC), electrolyte leakage (EL), chlorophyll ‘a’ fluorescence and gas exchange (CO_2_ assimilation, transpiration, stomatal conductance and intracellular concentration of CO_2_) were measured as described previously in detail by Kosmala et al. (2012) and Perlikowski et al. (2014) [5,8]. For all the physiological measurements, the second fully expanded leaves from the top of plant were used. RWC was calculated according to the following formula: RWC% = (FW−DW)/(SW−DW) × 100, where FW was leaf fresh weight, DW was leaf dry weight, and SW was leaf turgid weight. EL was measured using a conductivity meter (Hanna Instruments EC215 Conductivity Meter) and calculated as follows: L_1_/L_2_ × 100, where L_1_ and L_2_ were electrolyte leakage of fresh leaves and leaves frozen in liquid nitrogen, respectively. Chlorophyll ‘a’ fluorescence was measured with the HandyPEA fluorimeter (Hansatech Instruments Ltd., King’s Lynn, England) during midday. For RWC, EL, and chlorophyll ‘a’ fluorescence measurements three biological and technical replicates of all the analyzed genotypes at each time-point of experiment (C, D1, D2, D3, RH), were applied. Gas exchange parameters were measured through CIRAS-2 Portable Photosynthesis System (PP SYSTEMS) at three selected time-points (C, D3, and RH) in three biological and two technical replicates. A capacity for water uptake for each genotypes was measured throughout drought treatment.

### 5.4. Accumulation of Abscisic Acid

An accumulation level of abscisic acid was evaluated in pooled leaves using Plant hormone abscisic acid (ABA) Elisa Kit (CUSABIO, www.cusabio.com, CSB-E09159Pl). The analysis was performed according to manufacturer instructions in three biological replicates at each time-point.

### 5.5. Transcript Accumulation of the Calvin Cycle and Antioxidant Enzymes

RT-qPCR analyses were carried out for three enzymes of the Calvin cycle: plastid fructose-1,6-bisphosphate aldolase (pFBA), plastid phosphoglycerate kinase (pPGK), plastid glyceraldehyde-3-phosphate dehydrogenase (pGAPDH) and seven antioxidant enzymes: glutathione reductase (GR), chloroplastic glutathione peroxidase (GPX), chloroplastic Fe-dependent superoxide dismutase (Fe-SOD), chloroplastic Cu/Zn superoxide dismutase (Cu/Zn-SOD), manganese superoxide dismutase (Mn-SOD), L-ascorbate peroxidase (APX) and catalase (CAT). Total RNA was extracted from 100 mg of pooled leaves using the RNeasy Plant Mini Kit (Qiagen) according to the protocol. A remaining DNA was removed from the samples using the RNase-Free DNase set (Qiagen). cDNA was synthesized with the Maxima First Strand cDNA Synthesis Kit (Thermo Scientific). RT-qPCR assays were performed using the FastStart Essential DNA Probes Master (Roche) through the Bio-Rad CFX 96 thermal system as described by Pawłowicz et al. (2017) [6]. Reaction temperature profile was as follows: initial denaturation 95 °C for 10 min, followed by 44 cycles of 95 °C for 10 s, and 60 °C for 30 s and final 50 °C for 30 s. A relative quantification method (ΔΔCq) was used. Reactions were normalized using actin and ubiquitin as reference genes. Primer and TaqMan probe sequences of all the analyzed genes were designed based on the gene cDNA sequences through the Beacon Designer software (Table 2). All the measurements were carried out in three biological and two technical replicates at each time-point.

### 5.6. Protein Accumulation of the Calvin Cycle and Antioxidant Enzymes

Protein accumulation profiles of three enzymes of the Calvin cycle (pFBA, pPGK, pGAPDH) and seven antioxidant enzymes including glutathione reductase (GR, AS06 181), chloroplastic glutathione peroxidase (GPX, AS04 055), chloroplastic Fe-dependent superoxide dismutase (Fe-SOD, AS06 125), chloroplastic Cu/Zn superoxide dismutase (Cu/Zn-SOD, AS06 170), manganese superoxide dismutase (Mn-SOD, AS09 524), L-ascorbate peroxidase (APX, AS08 368) and catalase (CAT, AS09 501), were analyzed. Total proteins were extracted from pooled leaves using Hurkman and Tanaka protocol with slight modifications [4,62,63]. Briefly, 200 mg of powdered tissue was homogenized with 500 µL of extraction buffer (0.7 M sucrose, 0.5 M TRIS, 30 mM HCl, 50 mM EDTA, 2% DTT, and 0.1 M KCl). An equal volume of phenol was then added, vortexed and centrifuged at 21 500 g for 15 min. An upper phenol phase was transferred to new tubes and 500 µL of extraction buffer was added. After vortexing and centrifuging in the same conditions, proteins from phenol phase were precipitated by addition of 5 volumes of cold 0.1 M ammonium acetate in methanol in a new tube. After at least overnight incubation at −20 °C, samples were centrifuged at 9000 g at 0 °C for 30 min. The precipitate was washed once with cold ammonium acetate in methanol and twice in cold acetone, and dried in SpeedVac (Heraeus Instruments). Dried precipitate was dissolved in 150 µL of resolving buffer (50 mM TRIS, 2% SDS, DTT) at room temperature (RT) and then denatured for 5 minutes at 99 °C. Western blot assay was performed as described by Pawłowicz et al. (2012) [64]. Briefly, proteins were separated by 12% SDS-polyacrylamide gel and electroblotted (Trans-blot SD, Semi-Dry Transfer Cell, Bio-Rad) onto nitrocellulose membranes (Bio-Rad). Immunodetections of pFBA, pPGK, and pGAPDH were performed with the use of polyclonal antibodies (Agrisera) diluted at 1:4000. The specific antibody for pFBA (target peptide: TFEVAQKVWAETFYY) was produced earlier, as described by Perlikowski et al. (2016) [8], the specific antibodies for pPGK and pGAPDH were produced in this study based on the specific peptides used as antigens (target peptides: GITVTKADDVIGPEC and CNGKKITITSKRDPA, respectively). Protein levels of antioxidant enzymes were detected using the commercial rabbit polyclonal antibodies (Agrisera). The GR, GPX, Fe-SOD, Cu/Zn-SOD, Mn-SOD, and CAT antibodies were diluted at 1:4000, whereas the APX antibody was diluted at 1:2000. Membranes were incubated with the antibodies for 1 h. Antigen-antibody complexes were detected using a secondary anti-rabbit IgG-horse radish peroxidase conjugate (Sigma) in dilution 1:20,000 (1 h of incubation), chemiluminescent substrates Westar Supernova (Cyanogen) and ChemiDoc^™^Touch Igmagin System (Bio-Rad) to visualize the results. All the measurements were carried out in three biological replicates at each time-point.

### 5.7. Activity of Chloroplast Aldolase

An activity of pFBA in pooled leaves was measured according to the modified method of Sibley-Lehninger [65,66]. Chloroplast proteins were extracted as described by Kosmala et al. (2012) and Perlikowski et al. (2016) with slight modifications [5,8]. An amount of 1 g of frozen material was ground in liquid nitrogen and then suspended in 10 ml of chloroplast isolation buffer (CIB) (Sigma) with 0.1% BSA. Homogenized samples were filtered through a Sefar nitrex filter and centrifuged at 200 g at RT for 3 min. A collected supernatant was subsequently centrifuged for 15 min at 900 g at RT and then washed two times in 4 mL of CIB solution. Each time, the suspension was centrifuged at 900 g at RT for 15 min. A chloroplast pellet was dissolved in 500 µL of 0.1 M phosphate buffer (0.1 M Na_2_HPO_4_) with 3% Triton X-100, shaken by vortex and centrifuged at 21 500 g in RT for 10 min. A collected supernatant was used to determine the pFBA activity. A volume of 100 µL 0.06 M fructose-1,6-bisphosphate and 140 µL of incubation buffer (0.05 M 2,4,6-trimethylpyridine, 0.08 M hydrazine sulfate, 0.3 mM sodium iodoacetate) pH 7.4 was pre-incubated in water bath for 10 min at 30 °C. Additionally, a sample blank was performed for each biological replication which contained 100 µL of 0.06 M fructose-1,6-bisphosphate, 140 µL of incubation buffer and 300 µL of 10% trichloroacetic acid (TCA). A volume of 20 µL of chloroplast extract was added to pre-incubated solutions, mixed and incubated at 30 °C for 45 min. A reaction was stopped by adding 300 µL of 10% TCA to the solution, and tubes were chilled on ice. Ice-chilled samples were centrifuged at 21 500 g for 10 min in RT to remove precipitated proteins. A volume of 100 µL of each supernatant was incubated with 100 µL of 0.75 M NaOH at RT for 10 min and after that 100 µL of 0.1% 2,4-dinitrophenylhydrasine was added and samples were incubated for 10 minutes in a water bath at 30 °C. Tubes were taken out and 700 µL of 0.75 M NaOH was added to them and mixed well. After 3 min of incubation, absorbance at 540 nm was measured with reference to a sample blank. A standard curve was prepared with use of 0.01 mM D-glyceraldehyde as described in Perlikowski et al. (2016) [8]. Measurements were carried out in three biological and technical replicates at each time-point. An amount of produced trioses in the pFBA assay was read according to the standard curve and after calculations of glyceraldehyde mg produced by 1 g of plant sample during 1 h.

### 5.8. Activity of Antioxidant Enzymes

Activities of GPX, GR, SOD, and APX were assayed according to the manufacturer’s protocols by using Glutathione Peroxidase Assay kit (Abcam, ab102530), Glutathione Reductase Assay kit (Abcam, ab83461), Superoxide Dismutase Activity Assay kit (Abcam, ab65354) and Ascorbate Peroxidase Microplate Assay kit (Cohesion Biosciences, CAK1052), respectively. Plant extracts were prepared using pooled leaves. One unit of GPX activity was defined as the amount of enzyme that causes an oxidation of 1 µmol of NADPH to NADP^+^ under the assay kit condition per minute at 25 °C. One unit of GR activity was defined as the amount of enzyme that generates 1 μmol of nitrobenzoic acid (TNB) per minute at 25 °C. To measure SOD activity, an enzymatic extract was obtained by homogenization of 50 mg of leaf in ice cold 0.1 M Tris/HCl, pH 7.4 containing 0.5% Triton X-100, 5 mM β-Mercaptoethanol and 0.1 mg mL^−1^ phenylmethanesulfonyl fluoride. An inhibition of reduction of water-soluble tetrazolium salt (WST-1) which produces water-soluble formazan dye upon reduction by superoxide anion radicals, which are generated by xanthine oxidase and inhibited by SOD, was measured. One unit of SOD activity was the amount necessary to inhibit xanthine oxidation by 50% under assay conditions. One unit of APX activity was the amount of enzyme that oxidizes 1 μmol ascorbic acid per minute. Catalase (CAT) activity was determined after protein isolation from 0.2 g tissue homogenized in 1 mL of 50 mM KH_2_PO_4_ buffer, pH 7.0. The extract was centrifuged at 14 000 g for 20 min at 4 °C and the supernatant was collected. CAT activity was measured spectrophotometry according to Dhindsa et al. (1981) [67]. The activity of CAT was determined from the absorbance change at 240 nm based on the amount of decomposed H_2_O_2_. One unit of CAT activity was defined as the amount of enzyme catalyzing a decomposition of 1 umol H_2_O_2_ per minute calculated from the extinction coefficient 45.2 mM^−1^ cm^−1^. Bradford’s method was used to determine soluble protein content of samples [68]. The values obtained for each enzymatic activity measured were normalized using the soluble protein concentration and expressed per mg of protein. Activity measurements were performed on the Synergy HTX Multi-Mode Reader (BioTek) in three biological and two technical replicates at each time-point.

### 5.9. Lipid Peroxidation (TBARS Assay)

Lipid peroxidation was measured spectrophotometrically as a content in samples of thiobarbituric-reactive substances (TBARS) according to the method of Heath and Packer (1968) with slight modifications [69,70,71]. Briefly, 300 mg of second fully expanded leaves were homogenized with 2 mL of a buffer containing 0.25% TBA in 10% TCA at RT. After homogenization, samples were incubated at 100 °C for 15 min in a water bath. Next, samples were cooled on ice and centrifuged at 10,000 g by 10 min at 4 °C. The supernatant was collected and the absorbance was measured at λ = 532 nm and at λ = 600 in three biological and technical replicates at each time-point. An amount of TBARS was calculated through the following formula: TBARS (µM) = (A_532_ − A_600_)/155, where 155 was an extinction factor.

### 5.10. Production of Superoxide Anion Radical and Hydrogen Peroxide

Levels of superoxide anion radical (O_2_^•−^) and hydrogen peroxide (H_2_O_2_), were assayed spectrophotometrically. Superoxide anion radical measurement was performed according to Doke (1983), and Arasimowicz et al. (2009) [72,73]. Nitroblue tetrazolium (NBT) was used as a substrate which undergoes reduction by O_2_^•−^ to form diformazan. Discs from the second fully expanded leaves (0.6-0.8 cm in diameter) were incubated with 3 mL of a mixture containing 0.05 M potassium-phosphate buffer (pH 7.8) with 0.1 mM EDTA, 10 mM NaN_3_ and 0.05% NBT, for 1 h in the dark. Next, samples were heated at 85 °C by 15 min and cooled down on ice. The absorbance was measured at λ = 580 nm in three biological and technical replicates at each time-point. The level of O_2_^•−^ was expressed as the absorbance at 580 nm per 1 g of fresh weight (FW).

A concentration of hydrogen peroxide (H_2_O_2_) was assayed using the titanium (Ti4^+^) method [74,75]. An amount of 400 mg of plant tissue was homogenized on ice with 1.5 mL of 0.1 M potassium-phosphate buffer (pH 7.8). Obtained extracts were centrifuged at 14,000 g at 4 °C by 25 min. A volume of 1.5 mL of reaction mixture containing 400 μL of enzymatic extract, 600 μL of potassium-phosphate buffer and 500 μL of titanium reagent (0.6 mM PRL and 0.6 mM PTO in a ratio 1:1) were prepared for each sample. After 10 min of incubation, the absorbance was measured at λ = 508 nm in three biological and technical replicates at each time-point. The standard curve was used. The level of H_2_O_2_ was determined based on absorbance and expressed as µmol H_2_O_2_ per 1 g of fresh weight (FW).

### 5.11. Statistical Analysis

All the statistical analyses were performed with the STATISTICA 10.0 software (StatSoft, Tulsa OK, USA). A two-way analyses of variance (ANOVA), with genotype and time-point as classification factors, were performed. Differencesin physiological parameters, RT-qPCR analysis, protein accumulation, pFBA activity, TBARS assay, ROS measurement between theplants during experiment duration were evaluated using Fisher’s least significant difference (LSD) test at *p*-value 0.01. Homogeneity groups according to the test were denoted by the same letters on the graphs. A significance level of differences between the genotypes in WU was indicated by asterisks according to the student-*t* test at *p*-value 0.05.

## Figures and Tables

**Table 1:** Abbreviations of *F. arundinacea* and *F. glaucescens* genotypes used in the analyses and their definitions.

*F. arundinacea*	Fa-HDT	high drought tolerant
	Fa-LDT	low drought tolerant
*F. glaucescens*	Fg-HDT	high drought tolerant
	Fg-LDT	low drought tolerant

**Table 2:** Primers, probe sequences, and amplicon lengths of reference and target genes used for RT-qPCR analyses.

Gene	Primer Forward/Reverse	TaqMan Probe	Amplicon Length (bp)
Actin	GTCGAGGGCAACATATGCAA CCAGTGCTGAGCGGGAAT	TTCTCCTTGATGTCACGGAC	65
Ubiquitin	GCAAGAAGAAGACGTACA GACCTTGTAGAACTGGAG	CTTCACCTTCTTGTCCTTGTGCTT	86
pFBA	GAGACGTTCTACTACATG GAGGAGCTTGAGAGTGTA	TGTTCCTGTCCTTGCACTCGG	140
pPGK	CCTTGGTTGAGGAAGATAA CAGCAATGACAACATCAG	CTGGCAACAACTCTCCTGGC	102
pGAPDH	GTTCCAACACCAAATGTC TAGCCAAGAATGCCTTTC	CCTCTGATGCCGCCTTGATG	125
GR	GGGGAGTACGACTACGACCT TCGTAAGTCCACCCAAAGCC	GGCGGCGTCAGGGCCTCGCGCTT	254
GPX	TCACTCGGCGGCCTGGAGAA TTCACAGTGCGGGCTTACGA	CTACGCCACCGCCGCCACGGAGAA	212
APX	CTCGTATCGCAGGAGCTCG TTGGGCCACTCGCTAATGTT	CGGCTGCGGCTGGAGATGCGACGGC	188
Fe-SOD	TCTATCTCGGCGGTTCTCCA CCGTTGTTGTAGGCCTCCTT	GCTCGACACCAGCCCCTTCTACGGCCA	219
Cu/Zn-SOD	CCAGAGCATCCTCTTCGCC ATTGATGGAGGTGGAAGCCG	TCGCTCCGCCTCGTCTCCGCCCCC	287
Mn-SOD	TTGACGCCGCTGTCTCTAAGG TTTATCCAACGCCAGCCACA	GCTTCCGCCGTCGTCCAACTCCAGGGC	266
CAT	GTTCACCTTCCTCTTCGACG AAGTCGAACCTGTCCTCGTG	ACTACCGCCACATGGATGGCTCCG	297

## Supplementary Materials

Supplementary Materials can be found at https://www.mdpi.com/1422-0067/21/9/3174/s1. Additional files 1: Figure S1. Distribution of rDNA sequences in metaphase chromosomes of *F. arundinacea* (2n = 6x = 42) and *F. glaucescens* (2n = 4x = 28). Fa-HDT genotype of *F. arundinacea* (A); Fa-LDT genotype of *F. arundinacea* (B); Fg-HDT genotype of *F. glaucescens* (C); Fg-LDT genotype of *F. glaucescens* (D). 5S rDNA-red; 35S rDNA-green; chromosomes counterstained with DAPI-blue. Scale bars 5 µm. Additional files 2: Figure S2. Photographs presenting the phenotypes of *Festuca arundinacea* (Fa-HDT, Fa-LDT) and *F. glaucescens* (Fg-HDT, Fg-LDT) in the control conditions (C), on the 11th day of drought (D3) and after re-watering (RH). Additional files 3: Figure S3. Chlorophyll fluorescence parameters: ABS/RC, ABS/CS, TR_0_/RC, TR_0_/CS, ET_0_/CS, RC/CSm, RC/CS_0_, DI_0_/RC and DI_0_/CS in two genotypes of *F. arundinacea* (Fa-HDT, Fa-LDT) and *F. glaucescens* (Fg-HDT, Fg-LDT) before stress treatment (C), on the 3rd (D1), 6th (D2), and 11th (D3) day of water deficit and 10 days after re-hydration initiation (RH). The data represent means for nine individual measurements, error bars represent the standard errors (SE). Homogeneity groups are denoted by the same letters, according to Fischer LSD test (*p* = 0.01).

## Abbreviations

A	CO_2_ assimilation
ABA	abscisic acid
APX	L-ascorbate peroxidase
BSA	bovine serum albumin
CAT	catalase
*Ci*	CO_2_ concentration inside the leaves (interial)
Cu/Zn-SOD	chloroplastic Cu/Zn superoxide dismutase
DTT	DL-dithiothreitol
DW	leaf dry weight
E	transpiration
EDTA	ethylenediaminetetraacetic acid
EL	electrolyte leakage
Fe-SOD	Fe-dependent superoxide dismutase
FISH	Fluorescent in situ hybridization
FW	leaf fresh weight
g_s_	stomatal conductance
GPX	glutathione peroxidase
GR	glutathione reductase
GSH	reduced glutathione
HDT	high drought tolerant
L_1_	electrical conductivity of the fresh leaf exudate
L_2_	electrical conductivity of the leaf exudate immersed in liquid nitrogen
LDT	low drought tolerant
Mn-SOD	manganese superoxide dismutase
SDS	sodium dodecyl sulfate
SOD	superoxide dismutase
pFBA	plastid fructose-1,6-bisphosphate aldolase
pGAPDH	plastid glyceraldehyde-3-phosphate dehydrogenase
pPGK	plastid phosphoglycerate kinase
PPFD	photosynthetic photon flux density
PRL	4-(2′-pyridylazo)-resorcinol
PSII	photosystem II
PTO	potassium sodium tartrate tetrahydrate
ROS	reactive oxygen species
RT	room temperature
RWC	relative water content
TBARS	thiobarbituric-reactive substances
WU	water uptake
