# Supplementary figures and images for: Two Festuca Species—F. arundinacea and F. glaucescens—Differ in the Molecular Response to Drought, While Their Physiological Response Is Similar
Source: Int J Mol Sci. 2020 Apr 30;21(9):3174. doi: 10.3390/ijms21093174 (PMC7246586; doi:10.3390/ijms21093174)

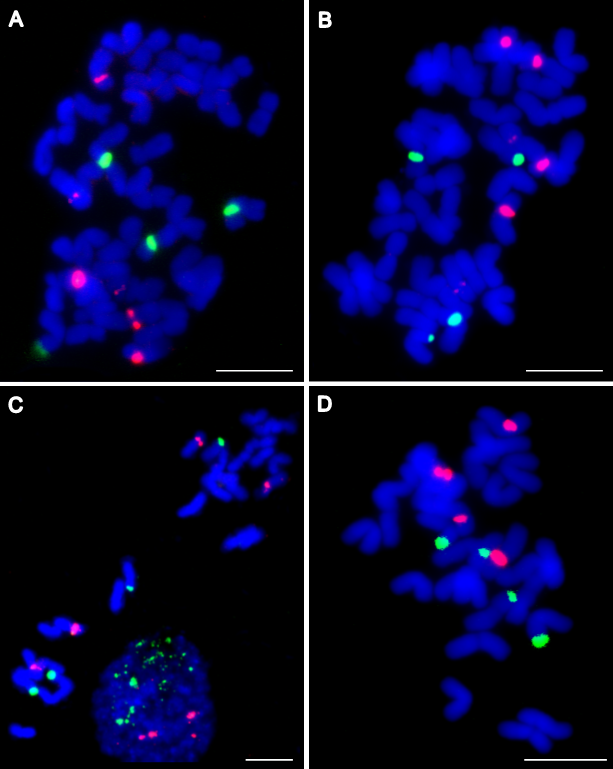

Supplement: Supplementary file 1 [file ijms-21-03174-s001.zip › ijms-790653-supplementary/Figure S1.tif]

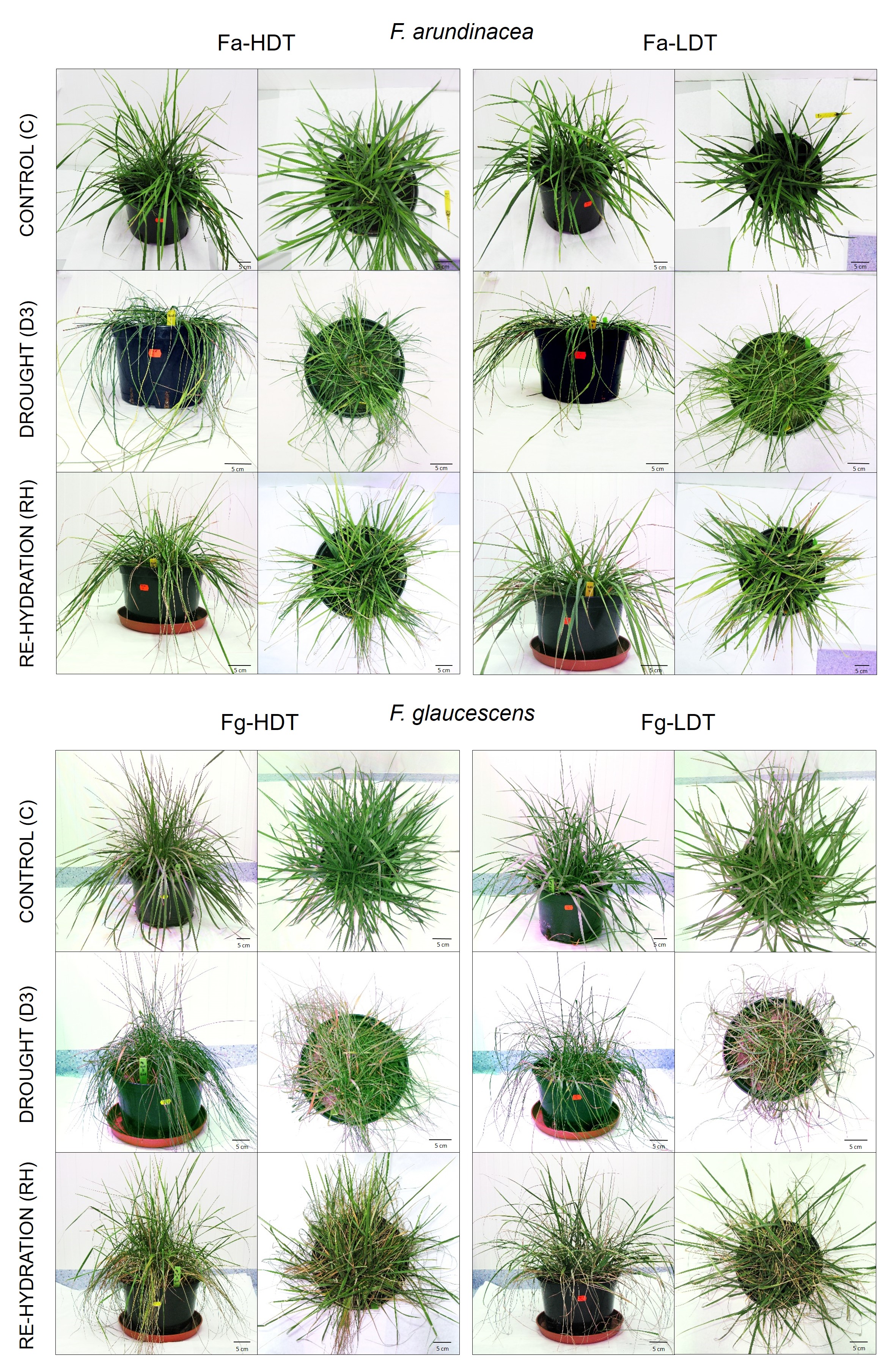

Supplement: Supplementary file 1 [file ijms-21-03174-s001.zip › ijms-790653-supplementary/Figure S2.jpg]

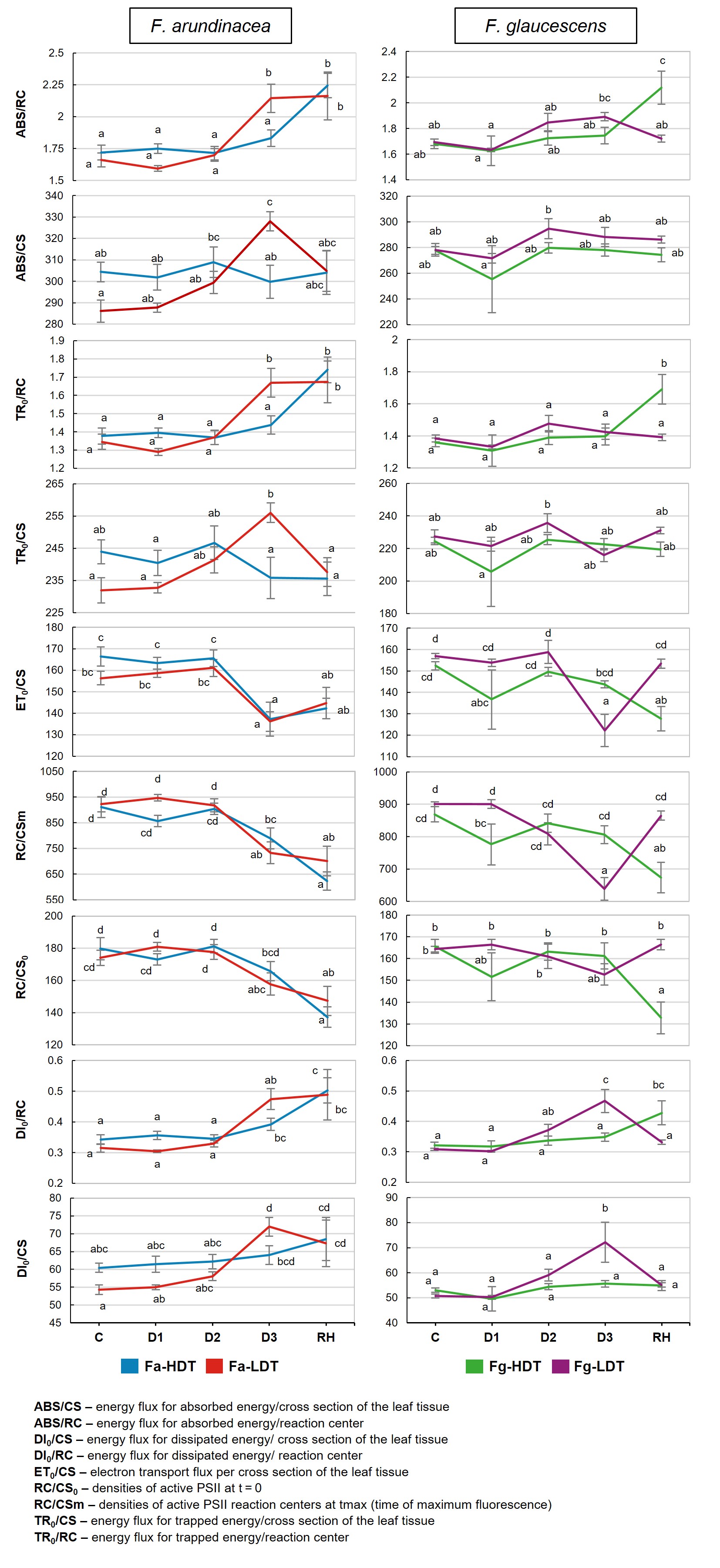

Supplement: Supplementary file 1 [file ijms-21-03174-s001.zip › ijms-790653-supplementary/Figure S3.jpg]
